# Graphene-Based Adsorbents for Arsenic, Fluoride, and Chromium Adsorption: Synthesis Methods Review

**DOI:** 10.3390/nano12223942

**Published:** 2022-11-09

**Authors:** Diego R. Joya-Cárdenas, Juliana P. Rodríguez-Caicedo, Armando Gallegos-Muñoz, Gabriela A. Zanor, Maya S. Caycedo-García, Cesar E. Damian-Ascencio, Adriana Saldaña-Robles

**Affiliations:** 1 Graduate Program in Biosciences, University of Guanajuato, Irapuato 36500, Mexico; 2 Department of Mechanical Engineering, University of Guanajuato, Salamanca 36800, Mexico; 3 Department of Environmental Engineering, University of Guanajuato, Irapuato 36500, Mexico; 4 Facultad de Ingenierías y Tecnologías, Instituto de Investigación Xerira, Universidad de Santander, Bucaramanga 680003, Colombia; 5 Department of Agricultural Engineering, University of Guanajuato, Irapuato 36500, Mexico

**Keywords:** graphene oxide, ions, adsorption, functionalization

## Abstract

Water contamination around the world is an increasing problem due to the presence of contaminants such as arsenic, fluoride, and chromium. The presence of such contaminants is related to either natural or anthropogenic processes. The above-mentioned problem has motivated the search for strategies to explore and develop technologies to remove these contaminants in water. Adsorption is a common process employed for such proposals due to its versatility, high adsorption capacity, and lower cost. In particular, graphene oxide is a material that is of special interest due to its physical and chemical properties such as surface area, porosity, pore size as well as removal efficiency for several contaminants. This review shows the advances, development, and perspectives of materials based on GO employed for the adsorption of contaminants such as arsenite, arsenate, fluoride, and hexavalent chromium. We provided a detailed discussion of the synthesis techniques and their relationship with the adsorption capacities and other physical properties as well as pH ranges employed to remove the contaminants. It is concluded that the adsorption capacity is not proportional to the surface area in all the cases; instead, the synthesis method, as well as the functional groups, play an important role. In particular, the sol–gel synthesis method shows better adsorption capacities.

## 1. Introduction

The availability of water is fundamental for life. However, the quality of water has decreased over the last years [1,2]. In places where surface water is scarce and has bad quality, underground water is the main source for industrial, irrigation, and drinking purposes. It is estimated that underground water is the main source for more than five hundred million people around the world [3,4]. The quality of underground water is influenced by a natural process such as the chemical interaction between soil, rock, and water, as well as anthropogenic activities [5]. The presence of contaminants in the water is a topic that has become more important over the last few years due to the negative effects on human health related to ions and heavy metals dissolved in it. For instance, the most common contaminants in the water reported in the literature are arsenite (As (III)), arsenate (As (V)), chromium (Cr (VI)), cadmium (Cd), copper (Cu), lead (Pb), fluoride (F${}^{-}$), and mercury (Hg). In particular, the As (III), As (V), F${}^{-}$, and Cr (VI) are considered the primary contaminants in water from either natural or anthropogenic activities [6,7,8].

Arsenic contamination has grown in recent decades, making it a growing concern, exposing millions of people to arsenic in the water around the world. For instance, As concentration higher than 10 μg L${}^{-1}$ is related to hyperpigmentation, hyperkeratosis, and cancer in different organs, which are known as arsenicosis [9], where the most common species in water are As (III) and As (V). On the other hand, fluorine contamination in water affects areas in developing and developed countries. Fluorine has a high electron affinity, which leads to the formation of fluoride ions. The fluoride concentration in water more than of 2 mg L${}^{-1}$ causes dental and/or skeletal fluorosis [10]. The potential risk to health due to the presence of chrome in groundwater is a concern around the world. Chromium’s toxic effects cause several diseases such as skin tumors in animals and damage the DNA by forming the Cr-DNA. The most common Cr ions are Cr (VI) and Cr (III), being 100 times more toxic Cr (VI) than Cr (III) [7]. To deal with the above-mentioned problems, several strategies were developed for the removal of harmful compounds in water, such as coagulation–flocculation, inverse osmosis, sedimentation, coagulation, and processes of chemical adsorption, among others [11,12,13,14].

In the last few decades, several adsorbents [2] to remove harmful compounds such as As (III), As (V), F${}^{-}$, and Cr (VI) on the water with specific capabilities were developed. The adsorption process is carried out through chemical and physical interactions between the sorbate and the adsorbent surface. In particular, graphene oxide (GO) is employed as an adsorbent in water and has been employed more frequently due to its semi-planar 2D structure with oxygenated functional groups, which are coupled by covalent bounds with the oxygen atoms over the carbon structure [15,16,17]. Among the functional groups, carbonile (C=O), carboxile (COOH), epoxy (C-O-C), and hydroxile (-OH) are found. These functional groups are important to provide adsorption sites as well as to increase the surface area, which is required to improve its adsorption capacities [18]. Thus, the wrinkles generated by the addition of functional groups always tend to increase the surface area and in this manner increase the possible sites of adsorption.

The advances in science and technology of adsorbent materials based on GO have shown their potential to remove heavy metals due to their excellent mechanical and electrical properties as well as physical characteristics such as surface area and laminar structure. Thus, the materials based on GO are a promising alternative to the removal of contaminants in water [19,20]. However, up to now, there are a few reports that provide useful information in regard to the synthesis techniques and their relation with the physicochemical properties of the materials as well as perspectives and trends of the novel materials based on GO for the removal of contaminants in water. Due to the above-mentioned, this review provides the advances, development, and perspectives of materials based on GO employed in the adsorption of single and several contaminants, such as As (III), As (V), F${}^{-}$ and Cr (VI) in water, providing a detailed discussion of the synthesis procedures as well the relationships between the synthesis procedure and its physical and chemical properties with a special emphasis on the better adsorbent materials developed so far. Finally, we provide a perspective on the promising trends in GO adsorption materials to remove contaminants.

## 2. Preparation and GO Characterization

### GO Preparation

GO can be synthesized in a dry and wet medium. In a dry medium, the oxidation reaction of graphene can occur through exposure to oxygen and its subsequent treatment with ozone under ultraviolet light [21]. On the other hand, in wet synthesis, the oxidation and exfoliation process occurs in an acid medium. These mechanisms were proposed by Brodie–Staudenmaier–Hummers [22].

**Figure 1:**
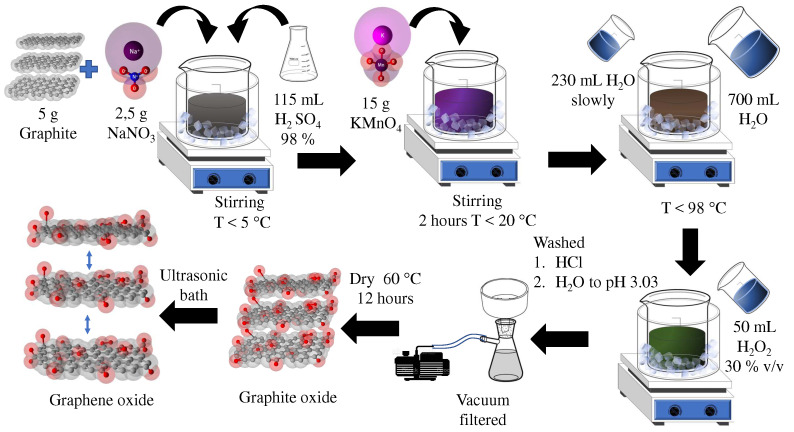
Synthesis of graphene oxide according to Hummer’s method modified.

Brodie made the first synthesis of GO by adding potassium chlorate to a suspension of graphite and nitric acid as fuming [23,24,25]. Staudenmaier improved this synthesis using a mixture of concentrated sulfuric acid and fuming nitric acid, gradually adding chlorate to the reaction, producing highly oxidized GO [26,27]. However, the syntheses proposed by Brodie and Staudenmaier generate toxic gases (NO${}_{x}$), representing an explosion risk in the reaction process. Hummers and Offeman proposed an alternative synthesis of GO under safe operating conditions. Figure 1 represents the GO synthesis according to the Hummer method, which consists of mixing graphite and NaNO${}_{3}$ in a 98% solution of H${}_{2}$SO${}_{4}$ under continuous stirring, keeping the reaction temperature around 5 °C using an ice bath. This is completed to integrate the oxidizing reagents into the graphite and thus avoid an abrupt reaction that allows the breaking of the graphene sheets. Then, potassium permanganate (KMnO${}_{4}$) is added, keeping the temperature below 20 °C, which is kept under continuous stirring for 2 h. After the first exfoliation stage, distilled water is added slowly and gradually, under constant temperature control, without exceeding 98 °C. Subsequently, distilled water is added again, and oxygen is added to the system by adding hydrogen peroxide H${}_{2}$O${}_{2}$ at 30% *v*/*v*, which produces an inactivation of the potassium permanganate action, oxidizing it to Mn${}^{4+}$. This solution remains at rest for 24 h to recover the graphite oxide, where it is washed with 5% HCl and then with H2O until reaching a pH of 3.03. Once the pH condition is met, the material is dried for 12 h at 60 °C, and exfoliation is performed to achieve the separation of the GO layers [28]. This synthesis achieves the same degree of oxidation reported by Stadenmaier; however, modifications were made to the Hummers method by adding oxidizing agents such as sodium nitrate (NaNO${}_{3}$) to obtain a greater presence of oxygenated functional groups between the interlaminar sites, thus increasing the distance between the graphite layer and decreasing the van der Waals forces. These modifications allow for an increase in the exfoliation of GrO to obtain GO through techniques such as ultrasound as well as mechanical agitation.

In the last few years, different GO syntheses have been developed using the modified Hummers method from commercial graphite, where variations are made to increase the number of oxygenated groups using different particle sizes, amounts of sulfuric acid, sodium dichromate, potassium permanganate, and sodium nitrate [22,29,30].

Other studies have proposed the synthesis of GO and reduced graphene oxide (rGO) by the chemical oxidation of graphite flakes using mixtures of sulfuric acid (H${}_{2}$SO${}_{4}$), phosphoric acid (H${}_{3}$PO${}_{4}$), and nitric acid (HNO${}_{3}$) as intercalating agents and potassium permanganate (KMnO${}_{4}$) and hydrogen peroxide (H${}_{2}$O${}_{2}$) as oxidizing agents [31]. On the other hand, to prevent the formation of toxic gases, NaNO${}_{3}$ has been used, making variations in the oxidation conditions and the proportions of the reagents to synthesize GO with different oxygen contents [32].

## 3. GO Composites for the Removal of Contaminants in Water

### 3.1. Arsenic

The synthesis of GO-based materials functionalized with various compounds such as goethite, ferrihydrite, zirconium, and gadolinium oxides employed for arsenic removal in water allowed the development of novel materials at a low cost. In the development of these compounds, GO is used as a support material to impregnate the functionalizing material [33,34,35,36,37,38,39,40,41]. Studies have been developed in the synthesis of GO by the modified Hummer method to increase the number of oxygenated groups as well as to improve the nucleation of the decorating materials. Among these developments is the hybrid calcium alginate nano adsorbent with GO microspheres (GO-MnO${}_{2}$-Goe-Ca-Alg), where it is found that GO does not play a direct role in the adsorption of arsenic. However, the GO is important in the formation of the nucleus-envelope type adsorbent, by increasing the specific surface area, allowing to obtain maximum adsorption capacities of 27.53 mg g${}^{-1}$ and 34.17 mg g${}^{-1}$, respectively, for As (III) and As (V), following the Langmuir isotherm model [33]. On the other hand, the coupling of ferrihydrite (MGOFE) as well as goethite (MGOGH) on GO favors the adsorption of As (V). In particular, MGOGH has reported an adsorption capacity of 66.88 mg g${}^{-1}$ at a temperature of 318 K and pH of 4.0. This increase in the adsorption capacity is mainly related to the experimental conditions as well as to the process of modification of the GO synthesized, and it suggests that the addition of NaNO${}_{3}$ oxidant agent during the synthesis increases the presence of oxygenated groups, which increase the interplanar distance in the basal plane [34].

The use of gadolinium oxide is particularly interesting due to its complexation, which is generated in the GO surface and the electrostatic attraction of arsenic ions and consequently reporting high adsorption capacities. Studies have been developed to evaluate the potential of empty sphere magnetic nanocomposites using gadolinium oxide in GO (GO-Gd${}_{2}$O${}_{3}$) and the incorporation of chitosan (CGO-Gd) for the elimination of As (V) from water. These materials showed adsorption capacities of 216.70 and 252.12 mg g${}^{-1}$, respectively, at pH 6.0 [40,41]. These studies suggest that the adsorption of As (V) for the modified materials with gadolinium oxide is mainly due to the chemical interactions as well as the electrostatic attraction of functional groups of carbon-containing oxygen with gadolinium oxide and the electrons of the π-π bounds.

Recently, other new adsorbent materials were developed by the functionalization of magnetic graphene oxide (MGO), which favors its later separation from the solution and thus increases the number of cycles in which the material can be reused. Moreover, the magnetization of GO allows stable dispersion since magnetite (Fe${}_{3}$O${}_{4}$) is uniformly dispersed in GO [42,43,44,45]. Some studies propose novel strategies for the use of MGO in the production of hydrogels (MGOH) and their application in arsenic removal. These materials (MGOH) show a maximum adsorption capacity of 25.1 mg g${}^{-1}$ for As (III) and 74.2 mg g${}^{-1}$ for As (V). It has been reported that the adsorption capacity is increased by having oxygenated groups along the hydrogel surface, and this benefits the microporous structure and superparamagnetic characteristics. In these materials, the magnetization values reach a specific saturation (Ms) of 57.8 emu g${}^{-1}$, which allows subsequent separation for reuse [42]. The incorporation of chitosan in the synthesis of MGO nanocomposites has allowed obtaining maximum As (III) adsorption capacities of 45 mg g${}^{-1}$. This increase in As (III) adsorption capacity is related to the formation of nanocomposites with a high surface area (about 152.38 m${}^{2}$ g${}^{-1}$) [46]. Similarly, the functionalization with polymers (MGO-IIP), ionic liquids (MGO-IL), and ethylenediamine (Fe${}_{3}$O${}_{4}$@GO-EDA) on the surface of magnetic graphene oxide has allowed modifying the surface and electrostatic charge of the adsorbents, increasing the adsorption sites for As (III) and As (V) and reaching adsorption capacities up to 160.65 mg g${}^{-1}$ for As (III) and 104.13 mg g${}^{-1}$ for As (V) [45,47,48].

GO reduction has been used as an alternative for the synthesis of new materials to improve the adsorption capacities of adsorbents [49,50,51,52,53]. For instance, syntheses have been carried out from GO nanocomposite partially reduced with magnetite (Mag-PRGO) adsorption capacity of 131.9 mg g${}^{-1}$ for As (V) with a pH ranging from 4.0 to 6.0 [50]. Priya et al. [51] by employing polymerization were able to synthesize nano adsorbents of reduced graphene oxide (rGO), with Fe-Al hydroxide coated with several concentrations of sodium alginate (FAH−rGO/SA), obtaining adsorbent materials dubbed FAH−rGO, FAH−rGO/SA−1 and FAH−rGO/SA−4 with adsorption capacities of As (V) of 115.39, 151.29 and 190.84 mg g${}^{-1}$, respectively. The high percentage in weight of sodium alginate (SA) in the FAH−rGO provides a surface area of 151.35 m${}^{2}$ g${}^{-1}$ for the FAH−rGO/SA−4 due to the intercalation of SA and FAH molecules in the GO surface, allowing a high adsorption capacity. The synthesis of Zr-MnO${}_{2}$@RGO nano compounds for the elimination of As (V) ions carried out by Yakout et al. [52] shows that the increase of surface area is quite important in the improvement of the adsorption capacity, as well as the affinity of the adsorption of rGO through the incorporation of Zr and MnO${}_{2}$. The experimental data obtained show a good agreement with the Langmuir isotherm being able to reach an adsorption capacity of 201.1 mg g${}^{-1}$ for As (V), reaching values higher than those obtained by Bobb et al. [50] and Priya et al. [51]. The above-mentioned arguments suggest that the performance of the nano compound is due to the bi-functionality with Zr and MnO${}_{2}$, which after its coupling with the nano compound was able to reach specific surface areas of 205.24 m${}^{2}$ g${}^{-1}$, modifying the characteristic planar layer structure of the GO by corrugating these layers.

The multifunctionalization of GO has been explored to improve the characteristics of adsorbents by providing specific properties, such as broad pH ranges, a specific affinity for contaminants, and an increase in adsorption active sites and specific surface area [36,54,55,56,57,58,59,60]. By a double functionalization of GO with ferric oxide and aluminum (GIAMO), Maji et al. [54] obtained an adsorption capacity of 42.28 mg g${}^{-1}$. This double functionalization allowed to increase the specific surface area to 190.54 m${}^{2}$ g${}^{-1}$, which was approximately 1.45 times higher than its precursor material (IAMO) 131 m${}^{2}$ g${}^{-1}$. Chen et al. [55] modified carboxylic graphene oxide (GO-COOH) with akageneite (β-FeOOH). The authors showed a wide pH range (from 3.0 to 10.0) and adsorption capacities of 77.5 mg g${}^{-1}$ for As (III) and 45.8 mg g${}^{-1}$ for As (V). This indicates that the surface functionalization of the hydrated GO, i.e., the addition of β-FeOOH on the GO-COOH surface, plays an important role due to the change of the surface charge of the composite material. In the same manner, Pervez et al. [61] developed a comparative study designed to examine the adsorption potential of the hybrid GO impregnated with quantum dots of goethite (α-FeOOH QDs@GO). These authors obtained the consecutive modulation of β-FeOOH with the organic ligands of acetate (Ac) and the GO compared with the GO-akageneite (β-FeOOH@GO) and akageneite (β-FeOOH), evaluating the role of the mediation of the environmental organic ligands. The maximum adsorption capacity of As (III) in α-FeOOH QDs@GO is 147.38 mg g${}^{-1}$, which is 2.52 and 4.60 times higher than the β-FeOOH@GO and β-FeOOH, respectively. In the same way, the maximum adsorption capacity of As (V) in β-FeOOH@GO is 69.03 mg g${}^{-1}$, which is 1.62 and 4.15 times higher than the α-FeOOH QDs@GO and β-FeOOH, respectively. The specific surface area and the proportion of micropores of α-FeOOH QDs@GO were 255.24 m${}^{2}$ g${}^{-1}$ and 41.4% respectively, and the pore diameter is reduced to 2.4 nm, which indicates that the presence of acetate reduces the particle size and facilitates the adsorption process. The surface area, the average pore diameter, and the pore volume for the β-FeOOH@GO reported were 202.60 m${}^{2}$ g${}^{-1}$, 5.12 nm and 0.36 cm${}^{3}$ g${}^{-1}$, respectively, which indicates a significant increase in the number of mesopores in comparison with the specific surface area of β-FeOOH with 141.06 m${}^{2}$ g${}^{-1}$. These results pointed out that the GO has a better dispersion and support to the β-FeOOH and as a consequence increases the adsorption capacity.

**Figure 2:**
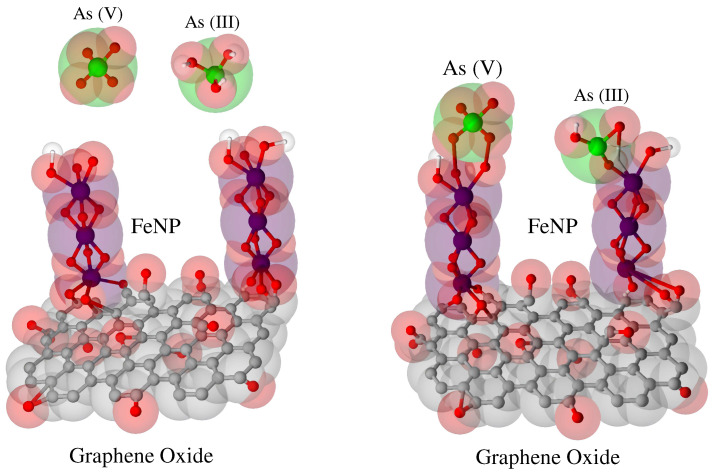
Possible mechanism of arsenic adsorption on FeNP@GO (figure adapted from Das et al., (2020) [62]). This proposed mechanism involves arsenic flux built up at the solution–adsorbent interface, which is followed by adsorption onto the iron nanoparticles (FeNPs) present on GFeN.

Iron–GO nanohybrid adsorbents (GFeN) have been developed by decorating structured iron nanoparticles on the GO FeNP@GO surface via the sol–gel process [62]. This material reported an adsorption capacity of 306 mg g${}^{-1}$ for As (III) and 431 mg g${}^{-1}$ for As (V). These very high adsorption capacities are held as a reference for the GO materials functionalized with iron. In addition to the improvements obtained in the adsorption capacities, this material obtained a specific surface area of 252.12 m${}^{2}$ g${}^{-1}$ (see Figure 2). These studies have shown a great advantage over the above-mentioned materials due to the short exposition times (around 10 min) required to remove 90% of arsenic. In summary, it is presented as an economic alternative due to its low cost and high adsorption capacity (>300 mg g${}^{-1}$). Table 1 summarizes the experimental data obtained from different studies in the Langmuir model for arsenic adsorption on functionalized GO compounds.

The above-mentioned materials allow inferring that the presence of oxygenated functional groups in the GO surface benefits both the chemical and electrostatic interactions between the adsorbent and the arsenic ions. They favor the coupling of several materials on the adsorbent surface, such as ferric materials and gadolinium oxides, which improves the adsorption capacity due to the modification of the porous structure, the area, and the adsorbent surface charge. In addition, the mechanisms which are employed in the synthesis affect the amount and type of functional groups on the GO surface as well as the affinity and changes its surface charge because of the nucleation, thus impacting the As (III) and As (V) adsorption capacity. For instance, among the materials reported in the technical literature, it is identified that the multi-functionalized materials have a wider pH range for their operation, with an adsorption capacity lower than 200 mg g${}^{-1}$, whereas the materials synthesized through the sol–gel method have the greatest adsorption capacity. In summary, the synthesis method plays an important role in the resulting adsorption capacity of the material. Among the materials with higher adsorption capacity were those reported by Das et al. [62] through the sol–gel methodology.

In summary, opposite to what can be inferred, the materials with the highest adsorption capacity are not the ones with the greatest surface area; instead, it is more related to the synthesis method employed as well to the functional groups on the surface.

### 3.2. Fluoride

In recent years, great efforts have been made to study GO-based materials with different functionalizations that have a higher fluorine adsorption capacity in aqueous systems [65,66,67,68,69]. Recently, some non-ferric adsorbent materials have been developed such as the one developed by Xu et al. [70]. These authors carried out a comparative study by the modification of nano-layers of GO with alumina GO/Al${}_{2}$O${}_{3}$ compared with the use of alumina γ-Al${}_{2}$O${}_{3}$ as adsorbent. The authors argue that the adsorption capacity of 4.68 mg g${}^{-1}$ reported for the GO/Al${}_{2}$O${}_{3}$ increases for the γ-Al${}_{2}$O${}_{3}$ which is found to be of 3.04 mg g${}^{-1}$. This increase is mainly related to the GO structure, which allows a surface area of 326.22 m${}^{2}$ g${}^{-1}$ which is slightly higher than that reported by the γ-Al${}_{2}$O${}_{3}$, which is 316.17 m${}^{2}$ g${}^{-1}$. In addition, the porous structure is modified due to the pore volume reduction for the GO/Al${}_{2}$O${}_{3}$, thus favoring the presence of active sites after the modification. In the same way, Prathibha et al. [71] developed an adsorbent material from sand coated with GO doped with zirconium (ZIGCS) [72], with a maximum adsorption capacity of 6.12 mg g${}^{-1}$. These authors propose a composite material by the addition of chitosan to a Zr (IV)-doped magnetic GO composite (Zr-MCGO) reporting an adsorption capacity of 8.84 mg g${}^{-1}$, reaching equilibrium after 1 h and 30 min. Zhang et al. [73] also employed an adsorbent based on a zirconium–chitosan membrane and GO (Zr-CTS/GO), and they obtained an adsorption capacity of 29.05 mg g${}^{-1}$. This is related to the coupling of Zr (IV) with oxygenated functional groups of the CTS/GO composite as well as the ionic interchange of Zr with the F${}^{-}$. Additionally, the GO improved the stability of the adsorbent and provides more adsorption sites for the Zr (VI). The increased adsorption capacity of these materials is mainly related to the Zr anchoring on the surface, which provides an increase in the active sites for the adsorption of F${}^{-}$ ions [71]. The mechanisms of fluorine adsorption on Zr-doped materials are mainly related to electrostatic forces, ligand exchange, and Lewis acid–base interactions [72,73].

The use of hydrogels for the encapsulation of GO-based functionalized materials has enabled the development of materials with great potential for water defluorination [67,68,69]. He et al. [64] proposed a material that consists of a hydrogel of sodium alginate and GO with a Yttrium basis Y-GO-SA1.0 through the sol–gel for the elimination of fluoride. This material shows a maximum adsorption capacity of 288.96 mg g${}^{-1}$ at a pH of 4 according to the Langmuir isotherm. This adsorption capacity is related to the amorphous pore structure developed by the GO by the hydrogel coupling.

**Figure 3:**
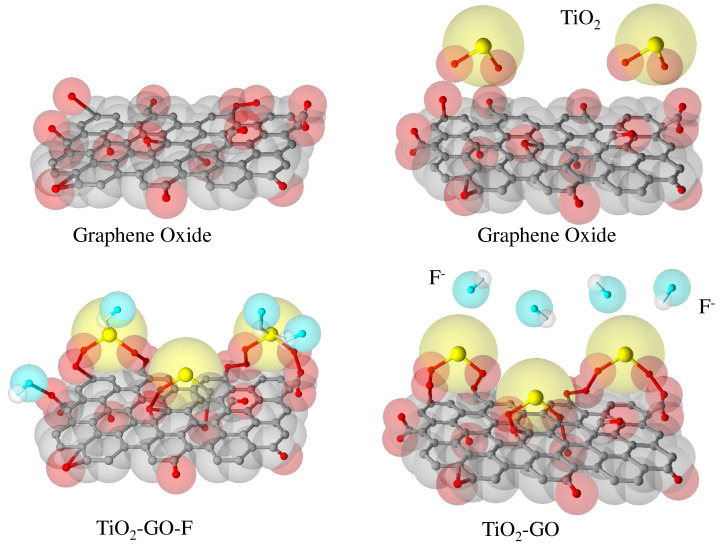
General representation of schematic synthesis of TiO${}_{2}$-GO nanocomposite and possible fluoride adsorption mechanism (figure adapted from Nehra et al. (2019) [74]). The schematic synthesis of TiO${}_{2}$-GO shows the nanocomposite and possible fluoride adsorption mechanism, and it describes the role of the hydroxyl groups in adsorption, producing an electrostatic attraction between the positively charged adsorbent and the negative F${}^{-}$ ion.

On the other hand, Nehra et al. [74] synthesized a nanocompound of pristine GO as recyclable adsorbent material of titanium oxide. The nanocompound (TiO${}_{2}$-GO) shows an adsorption capacity for fluoride ions of 342 mg g${}^{-1}$ with a specific surface area of 278 m${}^{2}$ g${}^{-1}$ according to the Langmuir isotherm, which is higher than that obtained by the TiO${}_{2}$, which is found to be of 78.4 mg g${}^{-1}$. These studies allow concluding that the coupling of different materials in the GO layers increases the adsorbent properties by the modification of the porous structure and increasing the surface area (see Figure 3).

Several authors have developed adsorbent materials by anchoring ferrous materials on graphene oxide due to their low toxicity, abundance on the earth and low cost [75,76]. An example of this is proposed by Kuang et al. [77], who synthesized a material by the functionalization of GO by the anchorage of goethite nanoparticles (FeOOH/GO) and akageneite (FeOOH+Ac/GO) through a hydrolysis procedure for the fluoride removal in an aqueous matrix. Thus, the addition of acetate as a chelator affects positively the nucleation of the ferric material, modifying the crystalline assembly. In particular, this chelator inhibits the growth of crystals, reducing the size of particles and increasing the macroscopic surface area, which leads to an increase in the adsorption capacity of fluoride with adsorption rates of 19.82 mg g${}^{-1}$. Fan et al. [78] synthesized regenerated GO nanocompounds (rGO) with the anchorage of goethite (α-FeOOH@rGO) through hydrolysis with ferric sulfate as a precursor material. This material has an adsorption capacity for fluoride of 24.67 mg g${}^{-1}$.

In the same manner, the compound of ferric oxide and aluminum coupled with graphene oxide (HIAGO) proposed by Kanrar et al. [79] shows fluoride adsorption of 27.8 mg g${}^{-1}$ due to the positive surface charge. This surface charge favors the electrostatic attraction of the fluoride ions since as pH is increased, there exists an increase in the mechanism of ion interchange. Mukhopadhyay et al. [80] obtained a composite material by applying a method of wet deposition to modify the ferric oxide (III) incorporating with Ce (IV) (CIHFO) and coupling with the hydrophilic GO (GO-CIHFO). The surface area of the GO-CIHFO composite is 189.57 m${}^{2}$ g${}^{-1}$, which is higher than the pristine CIHFO composite (around 140.71 m${}^{2}$ g${}^{-1}$), with an irregular surface morphology that consists of microcrystals of 2–3 nm in size and a mesopore structure of 3.54 nm. The GO-CIHGO composite shows a higher adsorption capacity of 136.24 mg g${}^{-1}$ in comparison with the adsorption capacity of GO (3 mg g${}^{-1}$) and the pristine CIHFO (32.62 mg g${}^{-1}$).

Other materials employed for the fluoride removal in water are the magnetic compounds of MgO/MgFe${}_{2}$O${}_{4}$, which are mounted in the GO substrate (MgO/MgFe${}_{2}$O${}_{4}$/GO) and nanocompounds of MgO/MgFe${}_{2}$O${}_{3}$ [81]. The MgO/MgFe${}_{2}$O${}_{4}$/GO has an adsorption capacity of 34 mg g${}^{-1}$ according to the Langmuir model with a specific surface area of 236 m${}^{2}$ g${}^{-1}$, which is higher than the MgO/MgFe${}_{2}$O${}_{3}$ with an adsorption capacity of 26 mg g${}^{-1}$ and specific surface area of 72 m${}^{2}$ g${}^{-1}$. This shows that the anchorage of this nanocompound in GO provides higher surface areas and smaller pore distribution, increasing the adsorption capacity.

In summary, it is concluded in a similar way to the As (III) and As (V) adsorption materials that the adsorption capacity depends on the surface properties as well as the chemical properties of the adsorbent. Some such properties include surface area, porosity, surface charge, and the variability and variety of oxygenated groups on the GO surface. All the above-mentioned properties depend directly on the synthesis methodology as well as the affinity of the materials coupled on the GO surface. Table 2 presents the adsorption capacity reported in the technical literature, which fits the Langmuir isotherm model. As observed, the highest adsorption capacity is related to materials with GO decorated with Y and titanium oxide. As observed, the specific surface area is not proportionally related to the adsorption capacity; instead, the synthesis method, as well as the functional groups, are the most important factors that affect the adsorption capacity.

### 3.3. Chromium

For the removal of chromium from water, various GO-based functionalized materials have been used to improve the adsorption capacity, including ferrous materials, materials with polymer modifications, materials with magnetic properties, materials with organic compounds, and even materials with reuse properties such as cellulose acetate [82,83,84,85,86,87,88]. To improve the mechanical strength and adsorption capacity, hybrid materials with GO based on non-ferrous composites have been developed with hydrotalcite (n-GO@HT) coupling chitosan (CS) to nano-graphene oxide (n-GO) through a hydrothermal method. This hybrid composite named n-GO@HTCS has obtained an adsorption capacity of 42.64 mg g${}^{-1}$ [89]. Materials with polymer nanofibers reported by Parlayici et al. [90] have been shown to be a low-cost alternative with high mechanical resistance. These authors synthesized a material by adding GO to Nailon-6.6 to produce nanofibers of N6.6/GO. The batch adsorption model for Cr (VI) is consistent with a maximum adsorption capacity of the Cr (VI) of 47.17 mg g${}^{-1}$. The nanostructured polymers such as polyaniline (PANI) have a high relation of surface area/volume and possess amine groups in their polymeric chain, which is considered a good adsorbent material of Cr (VI). Shaban et al. [91] studied the behavior of GO and PANI, employing it as an adsorbent of Cr (VI) in aqueous solutions. The adsorption behavior of GO and PANI is mainly controlled by the pH in the solution, achieving a better adsorption capacity under acid conditions with an adsorption capacity ranging from 49 to 59 mg g${}^{-1}$.

Another group of adsorbent materials is the magnetic compounds that are designed to effectively adsorb Cr(VI) through electrostatic attraction [92,93]. Ultrasonic radiation techniques have been used to synthesize GO nanocomposites by grafting with chitosan (CS-GO), obtaining adsorption capacities of 104.16 mg g${}^{-1}$ [94]. In the same way, comparative studies have been developed between the magnetic chitosan (Chi@Fe${}_{3}$O${}_{4}$) and the magnetic chitosan modified with GO (Chi@Fe${}_{3}$O${}_{4}$GO) to evaluate the potential of both materials as adsorbents of Cr (VI) from water [95]. The authors reported a maximum adsorption capacity of 142 and 100.51 mg g${}^{-1}$ for the Chi@Fe${}_{3}$O${}_{4}$ and Chi@Fe${}_{3}$O${}_{4}$GO, respectively, with a specific surface area of Chi@Fe${}_{3}$O${}_{4}$GO and Chi@Fe${}_{3}$O${}_{4}$ of 5.4 m${}^{2}$ g${}^{-1}$ and 2.3 m${}^{2}$ g${}^{-1}$, respectively, with a pore volume of 0.02 cm${}^{3}$ g${}^{-1}$ in both cases. Sheikhmohammadi et al. [96] employed a surface optimization methodology to optimize the adsorption of Cr (VI) in nanocompounds of magnetic GO (GO-Fe${}_{3}$O${}_{4}$) functionalized with two chelating ligands of phenazopyridine (GFP) and 2-mercaptobenzothiazole (GFM). The optimization of the Cr (VI) adsorption onto the GFM and GFP is carried out to increase the adsorption performance and minimize the adsorbent doses as well as to increase the initial concentration of Cr (VI). The optimal conditions are achieved with a pH of 6.55, an adsorbent dose of 0.098 g, a contact time of 178.4 min, and a concentration of Cr (VI) of 1 mg L${}^{-1}$ for GFP, whereas for a pH of 6.79 and adsorbent dose of 2.98 g L${}^{-1}$, there is a contact time of 118.6 min and a concentration of Cr (VI) of 4.41 mg L${}^{-1}$ for GFM.

**Figure 4:**
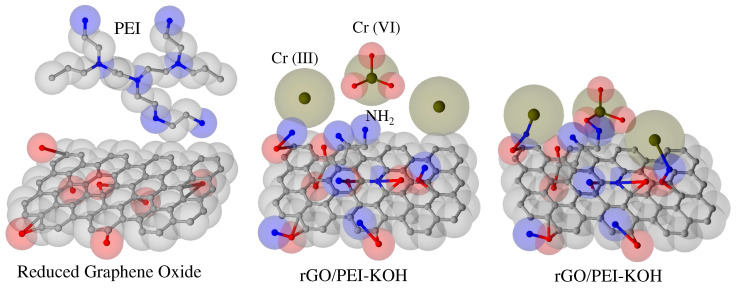
Synthesis of rGO/PEI-KOH and the adsorption process for the selective removal of chromium ions (figure adapted from [97]). The rGO/PEI-KOH synthesis and adsorption process for selective Cr (VI) removal and chelation between Cr (III) species and deprotonated amino groups (${}^{-}$NH${}_{2}$).

On the other hand, Tadjenant et al. [97] employed GO, PEI and KOH to synthesize nanocompounds of rGO (rGO/PEI-KOH) (see Figure 4). The resultant rGO/PEI-KOH adsorbent is highly effective for the removal of Cr (VI) at lower values of pH and reached an adsorption capacity of 398.9 mg g${}^{-1}$ following the Langmuir model. In the same way, Huang et al. [98] synthesized magnetic carbons co-doped with F and N (FNMC) through a method of simple pyrolysis, and the adsorbent is employed for the elimination of Cr (VI) in aqueous solutions. Since the charge density on the surface of the adsorbent plays an important role in the removal of Cr (VI), it was carried out the codification of N and F, which increases the negative density charge in the adsorbent surface. The synergic effect between the F and N dopants onto the magnetic carbon for the removal of Cr (VI) shows an adsorption capacity of 188.7 mg g${}^{-1}$ in a neutral solution, whereas the acid solution has an adsorption capacity of 740.7 mg g${}^{-1}$. This allows concluding that the more effective materials developed based on GO for the removal of Cr (VI) are related to the ones with negative charge surfaces, which benefits the adsorption process. These favorable adsorption properties under acidic conditions are related to the higher electronegativity of the fluorine for the nitrogen atoms, allowing a better ability to modulate the negative density charge of the adsorbent. Thus, it is suggested that the addition of two kinds of heteroatoms endow electronic features that favor adsorption.

Seeking to combine the advantages of GO as well as increase the specific surface area of materials for Cr (VI) removal in aqueous solutions, Wu et al. [99] synthesized a composite of FeS and GO dubbed GO/FeS. The results indicate that FeS nanoparticles effectively decorate the GO surface through -OH, C-O, C=O, and O=C-O functional groups. The maximum adsorption capacity for Cr (VI) adsorption obtained by the Langmuir model is 138.5 mg g${}^{-1}$, which is favored under acidic conditions. Similarly, bio-nanocomposite hybrid film materials based on GO/fungal hyphae (GO-FH) interaction have been developed. The results indicate that the adsorption of Cr (VI) on GO-FH material depends only on pH, with a maximum adsorption capacity of 212.76 mg g${}^{-1}$ and a pH of 2.0 according to the Langmuir isotherm [100].

Other materials have been developed with different strategies such as the fabrication of 3D magnetic hydrogels loaded with rGO. In this material, cellulose-bound magnetic nanoparticles (MP@cellulose) are synthesized by chemical coprecipitation and loaded together with rGO into poly(ethylene glycol) dimethacrylate-based hydrogels during fabrication by photopolymerization. The addition of poly(ethyleneimine) (PEI) allows the selective removal of Cr (VI) with an adsorption capacity of 313 mg g${}^{-1}$ [101].

Table 3 below presents the experimental data obtained from different studies in the Langmuir model for chromium adsorption on functionalized GO compounds.

In summary, in contrast to the previous sections for the As (III) and As (V) and F${}^{-}$ adsorbents where the adsorption capacity does not depend on the surface properties as well as the adsorbent chemical properties, for the chromium adsorbents, we observed a proportional relation between the adsorption capacity and the specific surface area. All the above-mentioned properties depend directly on the synthesis methodology as well as the affinity of the materials coupled on the GO surface; however, the number of works that report the specific surface area is not enough to generalize this conclusion. Table 3 presents the adsorption capacity reported in the technical literature, which fits the Langmuir isotherm model. Among the Cr (VI) adsorbents, the materials synthesized through magnetic hydrogels stand out. However, in contrast with the As(III) and As (V) and F${}^{-}$ adsorbents, the Cr (VI) adsorbents based on non-ferric composites show a high adsorption capacity as reported by Huan and Tadjenant where the selective removal of chromium ions is the most predominant feature.

### 3.4. Multicomponent Adsorption

The removal of several contaminants in water has driven the engineering of different strategies which allow removing simultaneously all the contaminants. One of these strategies has been proposed by Pal et al. [102], where a nanocomposite membrane is synthesized based on graphene through interfacial polymerization (IP), with the chemical union of the graphene oxide layer (GO) to the polyethersulfone surface. The membranes applied in a fixed module of a cross-flow planar layer can remove more than 98% of arsenic and around 80% of fluoride in contaminated water. On the other hand, the work reported by Verduzco et al. [103] employs compacted graphene composites (GCs) for the As (V) and Cr (III) removal of contaminated water by electrodeposition, where maximum adsorption capabilities of 38 mg g${}^{-1}$ and 32 mg g${}^{-1}$ for the As (V) and Cr (III) ions, respectively, were obtained. The As (V) and Cr (III) ions are removed in drinking water, and we found an increase in the percentage of removal from 70% to 87% for As (V) and 70% to 98% for Cr (III), which indicates that the additional ions of drinking water favor the performance of graphene composites for the elimination of contaminants.

Other studies developed for the removal of As (V) in water employed an analysis of adsorption and co-adsorption as reported by He et al. [64]. In this work, we employed a hydrogel GO–sodium alginate immobilized with yttrium (Y-GO-SA) to remove arsenic and tetracycline (TC). In a single system, the adsorption of As (V) and TC depends mainly on the pH of the solution, with an optimum pH of 5.0 for As (V) and 7.0 for TC. The adsorption equilibrium is obtained after 32 h for As (V) and 40 h for TC. The co-adsorption of As (V) and TC in the binary system indicates that the presence of TC suppresses the adsorption of As (V) due to the competition of active sites, whereas the effect of As (V) in the adsorption of TC is negligible due to the equilibrium: namely, it improves the anion-π interaction and reduces the Y ion competition in the adsorption of TC. Other works implemented materials for the removal of As (V) together with another contaminant as developed by Sadeghi et al. [63]. These authors, based on the GO nanoribbons hypothesis (GONR) as unidimensional graphene with more desirable borders as well as higher specific surface area than other carbonaceous nanomaterials, were able to obtain more functional groups containing oxygen in its borders and basal planes and thus having a higher adsorption capacity. This research synthesized GONR by decompressing carbon nanotubes in the multiple walls (MWCNT), and it evaluated the behavior of the adsorption through the assisted elimination by ultrasound of As (V) and Hg (II) ions in an aqueous solution. The results show that the As (V) ions are adsorbed more easily than the Hg (II) ions obtaining adsorption capacities of 155.61 and 33.02 mg g${}^{-1}$ for As (V) and Hg (II), respectively.

Table 4 below presents the experimental data obtained from different studies for the multicomponent adsorption on functionalized GO compounds.

Therefore, few studies related to the multi-adsorption of contaminants are found in the technical literature. In general, the literature reports the removal percentage instead of the adsorption capacity and its behavior in ion selectivity and competition situations reflecting a high affinity for some of the contaminants. For instance, in cases where Hg (II) and As (V) compete, the adsorbent materials show a higher affinity to As (V), and as a consequence, the As (V) removal percentage is higher than the Hg removal percentage. Thus, it is observed as a necessity to develop and study the behavior of adsorbent materials in situations of ion competition, since it is a necessity that occurs in real situations.

## 4. Conclusions

This review shows the advantages in the development of adsorbent materials based on GO, which has shown great efficiency in the removal of several contaminants, such as As (III), As (V), F${}^{-}$, and Cr (VI). These properties are mainly related to the complexity of the GO surface, as well as its affinity and electrostatic attraction, the mechanisms of ionic interchange, and the availability to be coupled with different materials, which improve its adsorption capabilities, selectivity, and specific properties such as surface charge and porosity. The functionalized materials based on GO can be mainly classified into three groups: namely, non-ferric materials, ferric materials, and materials synthesized through sol–gel. It is observed that the adsorption capacity is not directly related to the specific surface area; instead, the synthesis method as well as the functional groups play a more important role. For instance, the As (III) and As (V) adsorbent materials synthesized through the sol–gel methodology reported by Das et al. [62] have the greatest adsorption capacity, whereas the ferrous material with the highest specific surface area is reported by Su et al. [37]. The main difference between these two materials is related to the oxidation process carried out by Su et al. through the Hummers method, which provides the material with a mesopore structure, whereas the sol–gel provides many sites for adsorption. In the case of F${}^{-}$ adsorbent materials, similar behavior is observed: for instance, the ferric material reported by Kuang et al. [77] has the highest adsorption capacity, whereas the material reported by Nehra et al. [74] synthesized through a hydrothermal method has the highest adsorption capacity. On the other hand, the multi-functionalized materials adsorbed the contaminants in a large range of pH, whereas the single-functionalized materials work better at specific pH conditions. So, the multi-functionalized materials are more robust to applications with varying pH or in conditions where it is not possible to control the pH in the aqueous solution.

## 5. Future Perspective

In general, we observed a tendency toward the nucleation of different compounds such as iron, magnesium, and titanium on the GO surface, allowing improving the above-mentioned characteristics, in addition to the affinity toward different contaminants such as As (III), As (V), F${}^{-}$ and Cr (VI) simultaneously, which suggests that a prolific research topic could be related to the engineering of GO-based adsorbents functionalized and evaluated in multicomponent systems with specific capabilities at lower cost and their implementation on an industrial scale.

## Figures and Tables

**Table 1:** Arsenic adsorption on GO nanofunctionalized composites.

Composite	Maximum Adsorption Capacity (mg g${}^{-1}$)	pH	BET Surface m${}^{2}$ g${}^{-1}$	Reference
MGOGH	66.88 As (V)	5	287	[34]
Sn${}_{0.95}$Fe${}_{0.05}$O${}_{2-\delta }$	105 As (III)	6–8	135	[36]
GO/CuFe${}_{2}$O${}_{4}$	51.64 As (III)	7	-	[56]
	124.68 As (V)			
Mag-PRGO	131.9 As (V)	4–6	95.7	[50]
α-FeOOH QDs@GO	147.38 As (III)	2–11	255.24	[61]
	69.03 As (V)			
FeO${}_{x}$–GO-80	147 As (III)			[37]
	13 As (V)	6.5	341	
GONR	155.61 As (V)	6	-	[63]
MGO-IL	160.65 As (III)	7	-	[48]
	104.125 As (V)			
FAHrGO/SA-1 FAH-rGO/SA-4	151.29 As (III)	2–11	151.35	[51]
	190.84 As (V)			
Zr-MnO${}_{2}$@RGO	201.1 As (V)	4	205.24	[52]
GO-Gd${}_{2}$O${}_{3}$	216.70 As (V)	6.0	50.91	[40]
CGO-Gd	252.12 As (V)	6.0	61.28	[41]
Y-GO-SA	273.39 As (V)	5	-	[64]
FeGN@GO	306 As (III)			[62]
	431 As (V)	7	252.12	

**Table 2:** Fluoride adsorption on composites of GO.

Composite	Maximum Adsorption Capacity (mg g${}^{-1}$)	pH	BET Surface m${}^{2}$ g${}^{-1}$	Reference
α-FeOOH@rGO	24.67	3–12	-	[78]
HIAGO	27.8	5.5–7	-	[79]
FeOOH+Ac/GO	19.82	2.75–10.8	255.24	[77]
Zr-CTS/GO	29.05	3–11	1.6	[73]
MgO/MgFe${}_{2}$O${}_{4}$/GO	34	6.8	236	[81]
GO-CIHFO	136.24	7	189.57	[80]
Y-GO-SA1.0	288.96	4	147	[64]
TiO${}_{2}$-GO	342	6	278	[74]

**Table 3:** Chromium adsorption on composites of GO.

Composite	Maximum Adsorption Capacity (mg g${}^{-1}$)	pH	BET Surface m${}^{2}$ g${}^{-1}$	Reference
N6.6/GO	47.2	2	-	[90]
GO	49.0	3	-	[91]
PANI	59			
MoS${}_{2}$/rGO	80.8	2	79.35	[87]
GO/FeS	138.5	4	-	[99]
RGO/NiO	198.0	4	-	[88]
GO-FH	212.7	2	-	[100]
RGO/PEI/Fe${}_{3}$O${}_{4}$	266.6	-	-	[92]
GCF	270.3	2	-	[93]
MP@celulose-GO	313.0	-	-	[101]
rGO/PEI-KOH	398.9	3	-	[97]
FNMC	740.7	3	124.6	[98]
Chi@Fe${}_{3}$O${}_{4}$	142.0	2	5.4	[95]
Chi@Fe${}_{3}$O${}_{4}$GO	100.5	2	2.3	

**Table 4:** Multicomponent adsorption on composites of GO.

Composite	Maximum Adsorption Capacity (mg g${}^{-1}$)	pH	BET Surface m${}^{2}$ g${}^{-1}$	Reference
GCs	38 As (V)	6	47.63	[103]
	32 Cr (III)			
Y-GO-SA	273.39 As (V)	5	147	[64]
	477.9 TC	7		
GONR	155.61 As (V) 33.02 Hg (II)	6	-	[77]

## Data Availability

Not applicable.
